# Testing the Stem Dominance Hypothesis: Meaning Analysis of Inflected Words and Prepositional Phrases

**DOI:** 10.1371/journal.pone.0093136

**Published:** 2014-03-27

**Authors:** Minna Lehtonen, Gabor Harrer, Erling Wande, Matti Laine

**Affiliations:** 1 Cognitive Brain Research Unit, Cognitive Science, Institute of Behavioural Sciences, University of Helsinki, Helsinki, Finland; 2 Department of Psychology and Logopedics, Abo Akademi University, Turku, Finland; 3 Department of Finnish, Stockholm University, Stockholm, Sweden; University of Leicester, United Kingdom

## Abstract

We tested the hypothesis that lexical-semantic access of inflected words is governed by the word stem. Object drawings overlaid with a dot/arrow marking position/movement were matched with corresponding linguistic expressions like “from the house”. To test whether the stem dominates lexical-semantic access irrespective of its position, we used Swedish prepositional phrases (locative information via preposition immediately preceding the stem) or Finnish case-inflected words (locative information via suffix immediately following the stem). Both in monolingual Swedish and in bilingual Finnish-Swedish speakers, correct stems with incorrect prepositions/case-endings were hardest to reject. This finding supports the view that the stem is indeed the dominant unit in meaning access of inflected words.

## Introduction

Morphemes are the smallest meaningful units in language. With a relatively small number of morphemic elements it is possible to create large numbers of complex words that bear systematic mappings between form and meaning. Psycholinguistic models of morphological processing vary with respect to the emphasis they place on morphemic units in word recognition and production, and the level of processing at which these units may come into play (e.g., [1]–[7]).

In the Finnish language, lexical decision experiments [4], [8]–[10], eye-movement registrations [11] as well as data from patients with acquired aphasia [12], [13] have shown that most inflected nouns elicit a processing cost (e.g., longer reaction times or fixations, higher error rates) when compared to matched monomorphemic nouns. This robust effect has been taken as evidence that the majority of Finnish inflected words are decomposed into a stem and a suffix during recognition. According to Niemi et al. [4], morpheme-based representations are activated for inflected words both at an early visual word form level as well as at a later semantic-syntactic level. Behavioral and brain imaging studies investigating the functional locus of the processing cost related to Finnish inflections have shown that this cost primarily stems from the later semantic-syntactic level [14]–[17], i.e., the access and integration of the meaning of the stem and suffix of the inflected word.

Much of recent psycholinguistic evidence in other languages also supports the notion that morpheme-based representations are activated at least initially during recognition of morphologically complex words (see, e.g. [5], for a review). Most masked priming studies, using very short prime presentation times, have shown that semantic information is not accessed at early stages of word recognition ([5]; but see, e.g., [3], for a different view). At later stages of processing, however, semantic properties are assumed to be activated. For instance, effects in priming studies with longer prime presentation times or with a cross-modal setup are modulated by the semantic relationship between the prime and the target (e.g., [18]–[20]. With regard to theories concerning morphological processing, Schreuder and Baayen [6] proposed an interactive activation race model that assumes that after access representations, morphemes (or whole words) activate concept nodes that are connected to their semantic and syntactic representations. Morphological segmentation at access level is assumed to be followed by semantic activation and integration and syntactic licensing of the constituents. Meunier and Longtin [20] propose a framework in which morpho-orthographic segmentation is followed by a morpho-semantic level where the combinability of the morphemes is assessed and the constituents are semantically integrated. Taft & Nguyen-Hoan [7], in turn, assumed an intermediate lemma level between form and meaning.

While most models of morphological processing include a semantic level in their architecture, the majority of recent studies have focused on earlier stages of processing, the prelexical/form level (e.g., [5]) or the lemma level (e.g., [7]). The question of semantic access in inflected words has rarely been directly addressed experimentally. In the visual modality, access to the meaning of the morphological constituents of transparent affixed words, such as inflections, could take place either via the stem or via the stem and affix in parallel. The stem is likely to bear important content information whereas affixes typically convey rather systematic grammatical and/or semantic information. Affixes are often elements with a very high frequency in the language, and this feature may boost their role in the access process [21]. Laine [22] employed a semantic decision paradigm to investigate whether the meaning of an inflected word is primarily accessed via the stem, or via the stem and suffix in parallel. Participants saw a picture and were then asked to assess whether the picture matched an inflected written word with respect to the object and the spatial information present in both the picture and the inflected Finnish word (e.g. *talo+sta*: ‘house + ‘from’  =  ‘from the house’). It was found that non-matching items carrying the correct stem were clearly hardest to reject. In contrast, non-matching items carrying the correct suffix did not cause interference in semantic decision. This finding supports the view that at the semantic level, suffix-related information is secondary to the stem and it becomes available later than stem-related semantic information.

In Finnish the inflectional locative case marker is always a suffix. Therefore, the finding of Laine [22] supporting stem-governed access may be confounded by the position of the case marker in a word, i.e., being potentially available later in left-to-right reading. For instance, it has been shown that prefixed morphologically complex words do not show similar cumulative base frequency effects to suffixed words [23]. Moreover, word onsets have been shown to be psychologically more salient than other parts of the words (see, e.g., [24], for an overview). Consequently, affix priming effects have sometimes been shown to be stronger for prefixed than suffixed words in both masked and overt priming (e.g., [25] but see also [26], for significant suffix priming effects). It is therefore not certain whether the results of Laine [22] reflect the dominance of the stem during access to affixed words, or whether they reflect the fact that the stem was the first (leftmost) unit in the word.

The locative cases used by Laine [22], despite being inflectional suffixes which often have a role in conveying syntactic relations in sentences, contain semantic information and can easily be depicted in images. A close comparison that circumvents the possible position confound is offered in Swedish which expresses such meanings by prepositions (“i”, “från”, “till”) that are placed directly prior to the noun. Here, we thus employed a similar experimental setup to that of Laine [22] with Swedish prepositional noun phrases carrying the same locative information but prior to the noun (*från huset*  =  ‘from’ + ‘house’ (*hus*) in definite form, ‘from the house’). We first tested Swedish monolingual speakers to see whether they also show the slowest response times to incongruent preposition but congruent noun, as the Finnish participants did with the incongruent case suffix but congruent stem. Second, in order to verify that the same participants show the effect in both languages, we tested Finnish-Swedish bilinguals with both the Finnish and Swedish versions of the task.

## Experiment 1

### Methods

#### Participants

The study had been approved by the Institutional Review Board of the Department of Psychology and Logopedics, Abo Akademi University, and all participants gave their written informed consent. The data of the two experiments will be available upon request. Twenty-three Swedish-speaking university students living in Sweden (13 females) volunteered for the experiment. Their mean age was 24.9 years (SD 4.3), and they had acquired only the Swedish language before the age of seven. They all had normal or corrected-to-normal vision and did not report any neurological illnesses.

#### Stimuli

**Figure 1:**
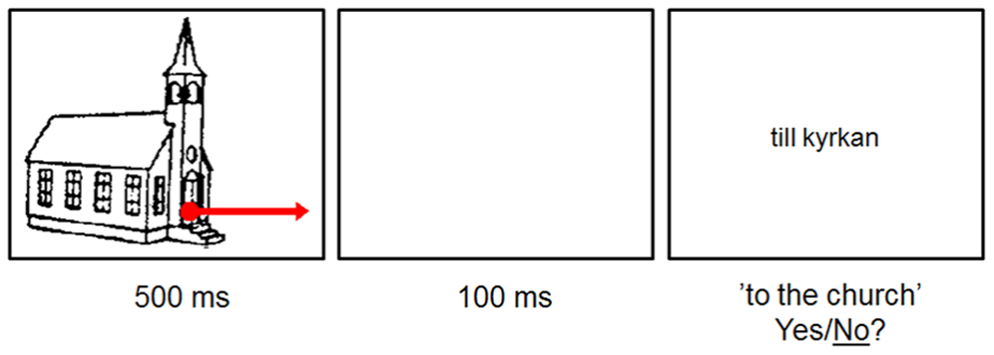
Trial structure of an item of the Swedish version of the picture-word form matching task.

For the target list, ten pictures representing common objects were selected. Three Swedish prepositions that carry locative information (corresponding to the English prepositions ‘in’, ‘from’, ‘to’) were added to the object names to form three different prepositional phrases for each object name. For example, for the target word *hus* ‘house’, the following prepositional phrases were constructed: *från huset*  =  ‘from the house’, *i huset*  =  ‘in the house’, and *till huset*  =  ‘to the house’. An example of stimulus pictures corresponding to the three prepositional phrases can be seen in Figure 1.

The stimulus list included altogether 160 picture-phrase pairs. It included the following target conditions: (a) 20 correct items where the picture is identical with the prepositional phrase, (b) 20 items where the noun was correct but the preposition was incorrect (N+P-), (c) 20 items where the noun was incorrect but the preposition was correct (N-P+), and (d) 20 items where both the noun and the preposition were incorrect (N-P-). Thus each object appeared twice in each of the abovementioned conditions, but with a different dot/arrow overlay and a different phrase. The occurrence of the three prepositions was more or less even within each condition (i.e., 7-7-6). In order to balance for the yes/no responses in the stimulus set, we included 80 filler stimuli (picture-phrase pairs) composed of 20 new object pictures, each appearing four times. Sixty of the fillers were correct (i.e., the picture corresponded to the subsequent prepositional phrase) and 20 were incorrect (7 N+P-; 7 N-P+; 6 N-P-). Finally, 24 practice stimuli (12 correct picture-phrase pairs) based on 4 objects not appearing in the experiment proper were designed.

#### Procedure

In the present cross-modal semantic decision task, each trial consisted of a picture followed by a prepositional phrase. The task of the participant was to determine whether the phrase matched with the picture just shown and press a corresponding button as quickly as possible. The picture depicted a concrete object that was overlaid by a red dot appearing with or without an arrow. The dot and the arrow position served to visualize location and movement of the dot (either inside the object, moving from the outside into the object, or moving from the inside of the object to the outside). Thus the object corresponded to the noun in the following prepositional phrase, while the dot and the arrow corresponded to the preposition (‘in’, ‘from’, ‘to’). The picture appeared on the screen for 500 ms, and after a 100 ms blank screen, the prepositional phrase appeared. The experiment was run by a special computer program (SuperLab Experimental Software, version 2.0, Cedrus Corporation), which recorded the participants' reaction times in milliseconds (the time from the appearance of the prepositional phrase to the response of the participant) as well as errors. Stimulus presentation was randomized separately for each participant.

The task was initiated by a practice block. At first, the participants saw the four practice objects and their corresponding names on paper to ensure that they recognized them correctly. To familiarize the participants with the experimental setup, all four object pictures had the red dots overlaid on them and one of the objects was shown with the dot-arrow combination corresponding to the phrases ‘from x’ and ‘to x’. After this familiarization and administration of written instructions, 24 practice trials were run. Before the experiment proper, the 30 object pictures (ten objects with three prepositions) used in the experiment were shown on paper, again with the corresponding name and overlaid by a red dot or a dot and arrow.

### Results

Prior to the analysis, incorrect responses and reaction times (RTs) that were more than 3 SD above or below the individual mean latency were discarded. We ran one-way analyses of variance with both subjects (F_1_) and items (F_2_) as random factors on RTs and error rates in the three non-matching (“incorrect”) conditions that are of particular interest here. We also performed the analyses (of both experiments) using linear mixed effects modeling with subjects and items as random factors. Using this approach, the pattern of results remained very similar to these ANOVAs (for more details of the linear mixed effects analysis results, see Supporting Information, File S1).

Analysis of RTs (see Table 1) revealed a highly significant effect of non-match stimulus type (F_1_(2, 44) = 37.64, p<.001; F_2_ (2, 57) = 28.8, p<.001). Pairwise comparisons by t-tests showed that the N+P- condition elicited significantly longer response latencies than the N-P+ condition (t_1_(22) = 5.46, p<.001; t_2_(38) = 5.67, p<.001) or the N-P- condition (t_1_(22) = 9.13, p<.001; t_2_(38) = 6.94, p<.001). The latter two conditions did not differ from each other (t_1_(22) = 1.31, p = n.s.; t_2_(38)<1).

**Table 1:** Mean RTs (ms) and error rates (%) of the Swedish monolinguals for the different conditions in Experiment 1.

Condition	RT (SD)	Error rate (SD)
N+P+	714 (116)	4.1 (4.8)
N+P-	880 (194)	5.7 (6.0)
N-P+	764 (145)	1.1 (2.1)
N-P-	747 (155)	1.1 (2.1)

The error rates (see Table 1) were low but one-way ANOVAs showed a highly significant effect of non-match stimulus type (F_1_(2, 44) = 11.46, p<.001; F_2_ (2, 57) = 13.22, p<.001). Subsequent pairwise comparisons indicated that the N+P- condition elicited significantly higher rates of errors than the N-P+ condition (t_1_(22) = 3.76, p = .001; t_2_(38) = 3.87, p<.001) or the N-P- condition (t_1_(22) = 3.43, p = .002; t_2_(38) = 3.87, p<.001). The latter two conditions had identical mean error rates.

### Discussion

The Swedish monolingual group showed a corresponding performance with the Finnish monolingual group of Laine [22]. The items with the correct noun and a non-matching preposition were harder to reject than the ones with an incorrect noun and a matching preposition, indicating that the noun is indeed the more dominant part of the phrase. This finding suggests that the results of Laine [22] with locative case markers are not confounded by the position of the suffix in the morphologically complex word. To provide further evidence, Experiment 2 was designed to verify that these effects can be observed in the same participants in both Swedish and Finnish.

## Experiment 2

### Methods

#### Participants

Nineteen bilingual Finnish-Swedish university students (15 females, mean age 24.3 (SD 5.1) years) volunteered for the experiment. They were neurologically healthy and had normal or corrected-to-normal vision. They had acquired both Finnish and Swedish in early childhood (before the age of seven), had continued using both languages since, and currently lived in a bilingual city in Finland. The bilinguals estimated their skills in both languages using a 6-point scale (0 =  no skills in the language, 6 =  perfect command of the language) and reported very similar values for both languages for speaking (mean for Swedish, 5.5; Finnish 5.4), speech comprehension (Swedish, 5.8; Finnish 5.9), reading (Swedish, 5.8; Finnish 5.8), and writing (Swedish, 5.1; Finnish 5.1). The bilinguals' language skills can thus be assumed to be quite balanced in Swedish and Finnish.

#### Stimuli

**Figure 2:**
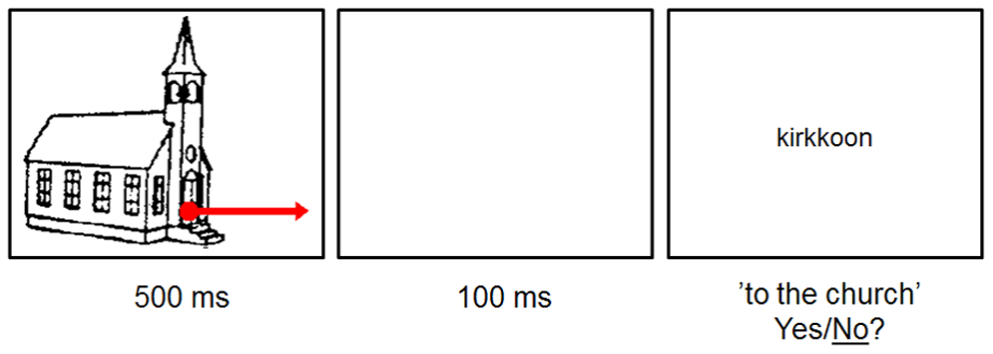
Trial structure of an item of the Finnish version of the picture-word form matching task.

The Swedish-language test of Experiment 2 was identical to that used in Experiment 1. The Finnish-language test was otherwise similar but employed case-inflected forms instead of prepositional phrases (see Figure 2). The three locative cases used, inessive, elative, and illative, correspond to the prepositions ‘in’, ‘from’, ‘to’. For example, for the target word talo ‘house’, the following case-inflected forms were constructed: talosta  =  ‘from the house’, talossa  =  ‘in the house’, and taloon  =  ‘to the house’. The target conditions thus were (a) 20 correct items where the picture was identical with the inflected word consisting of a noun stem + suffix (N+S+), (b) 20 items where the noun stem was correct but the suffix was incorrect (N+S-), (c) 20 items where the noun stem was incorrect but the suffix was correct (N-S+), and (d) 20 items where both the noun stem and the suffix were incorrect (N-S-). The same number of filler items and practice stimuli were included in the Finnish test as in the Swedish test.

#### Procedure

The Swedish-language and the Finnish-language tests were both conducted within the same testing session for each participant, and the presentation order of the tests was counterbalanced across the subjects. During the Swedish-language test, all communication was in Swedish, while during the Finnish-language test, all communication took place in Finnish. Within each test, the procedure followed that of Experiment 1.

### Results

The statistical analyses were carried out similarly to the Experiment 1. For *Swedish*, analysis of RTs (see Table 2) revealed a significant effect of non-match stimulus type both in the by-subject and by-item analyses (F_1_(2, 36) = 50.01, p<.001; F_2_ (2, 57) = 32.64, p<.001). Pairwise comparisons by t-tests showed that the N+P- condition elicited significantly longer RTs than the N-P+ condition (t_1_(18) = 6.49, p<.001; t_2_(38) = 5.92, p<.001) and the N-P- condition (t_1_(18) = 8.76, p<.001; t_2_(38) = 7.02, p<.001). The RT difference between the N-P+ condition and the N-P- condition was not significant (t_1_(18) = 2.04, p = .056; t_2_(38) = 1.45, p = .155).

**Table 2:** Mean RTs (ms) and error rates (%) of the Finnish-Swedish bilinguals for the different conditions in Experiment 2.

*Swedish*		
Condition	RT (SD)	Error rate (SD)
N+P+	656 (150)	2.6 (3.8)
N+P-	802 (156)	4.5 (6.9)
N-P+	691 (140)	2.1 (4.1)
N-P-	673 (138)	1.3 (3.6)

*Finnish*		
Condition	RT (SD)	Error rate (SD)
N+P+	695 (122)	1.8 (3.3)
N+P-	844 (149)	6.1 (5.0)
N-P+	682 (114)	1.3 (2.2)
N-P-	684 (130)	0.5 (1.5)

The error rates for Swedish (Table 2) did not differ between conditions in the subject analysis (F_1_(2, 36) = 1.76, p = .192) but showed a main effect of non-match stimulus type in the item analysis (F_2_ (2, 57) = 4.81, p = .012). Pairwise t-tests in the item analysis showed that the N+P- condition elicited significantly higher error rates than the N-P- condition (t_2_(38) = 2.73, p = .009), but the difference to the N-P+ condition was only marginally significant (t_2_(38) = 1.99, p = .053). The N-P+ condition did not differ from the N-P- condition in error rates (t_2_(38) = 1.00, p = .324).

The analysis for the *Finnish* RTs (see Table 2) also revealed a significant main effect of non-match stimulus type both in the by-subject and by-item analyses (F_1_(2, 36) = 56.89, p<.001; F_2_ (2, 57) = 56.34, p<.001). Pairwise t-tests showed that the N+S- condition elicited significantly longer response latencies than the N-S+ condition (t_1_(18) = 8.40, p<.001; t_2_(38) = 8.92, p<.001) and the N-S- condition (t_1_(18) = 8.40, p<.001; t_2_(38) = 8.42, p<.001). The latter two conditions did not differ from each other in their RTs (t_1_(18) = 0.16, p = .877; t_2_(38) = 0.24, p = .808).

The error rates for Finnish (Table 2) showed a significant effect of non-match stimulus type both in the by-subject and by-item analyses (F_1_(2, 36) = 14.89, p<.001; F_2_ (2, 57) = 14.09, p<.001). The N+S- condition elicited significantly higher error rates than the N-S+ condition (t_1_(18) = 3.51, p = .003; t_2_(38) = 3.56, p = .001) and the N-S- condition (t_1_(18) = 4.85, p<.001; t_2_(38) = 4.33, p<.001). The N-S+ condition did not differ in error rates from the N-S- condition (t_1_(18) = 1.14, p = .268; t_2_(38) = 1.24, p = .222).

We additionally tested whether these bilinguals performed similarly in both Finnish and Swedish using a two-way repeated measures ANOVA with factors language and non-match stimulus type. The results for RTs showed a significant main effect of non-match stimulus type (F_1_(2, 36) = 103.8, p<.001; F_2_ (2, 114) = 85.2, p<.001) but no significant main effect of language (F_1_(1, 18) = .273, p = .608; F_2_ (1, 114) = 1.48, p = .226) or an interaction between the two (F_1_(2, 36) = 2.73, p = .0.087; F_2_ (2, 114) = 1.83, p = .166). Similarly, the results for error rates showed a significant main effect of non-match stimulus type (F_1_(2, 36) = 11.37, p = .001; F_2_ (2, 114) = 17.9, p<.001) but no significant main effect of language (F_1_(1, 18) = 0; F_2_ (1, 114) = 0) or an interaction between the word type and language (F_1_(2, 36) = 0.785, p = .0.446; F_2_ (2, 114) = 1.56, p = .214). This indicates that the pattern of results was the same in both Finnish and Swedish.

### Discussion

The results of Experiment 2 showed that the congruent noun and mismatching preposition (N+P-) elicited clearly the longest RTs in Swedish, and so did the congruent stem and mismatching suffix condition (N+S-) in Finnish. In other words, it was more difficult to reject the phrase or word if the noun (stem) matched the picture, but this clearly did not depend on the position of the noun (stem). The Finnish-Swedish bilingual group thus displayed similar effects to the Swedish monolinguals in Experiment 1 in Swedish as well as to the participants of Laine [22] in Finnish. All in all, the present data confirm that the results obtained by Laine [22] were not confounded by the position of the stem.

## General Discussion

We focused on a less extensively studied but important aspect of morphological processing and tested whether semantic access to inflected words is governed by the stem, or whether access to the stem and suffix takes place in parallel. The study by Laine [22] was interpreted to support the former view but could have been confounded by the fact that the case marker is always a suffix in Finnish (i.e., the latter unit in left-to-right reading). Word onsets have been observed to be psychologically more salient than other parts of the words [24], suggesting that this earlier result might not depend on the nature of the stems and affixes per se but on the position of the morphemes in the word. The present study therefore utilized Swedish prepositions carrying the same locative information as the case-inflected words of Laine [22] but being positioned immediately prior to the noun. The results of both Swedish monolinguals and Finnish-Swedish bilinguals in both language versions of the task showed that it was significantly more difficult to reject a non-matching word or phrase when the noun/stem rather than the preposition/suffix was incongruent with the preceding picture. These findings support the notion that the stem is indeed the dominant element in the meaning access of inflected words, irrespective of its position in the word.

Different lines of evidence indicate that word stems are more influential units in word recognition than affixes. First, lexical decision studies have found that root frequency shows clearer effects in lexical decision than suffix frequency [27]. Second, semantic richness of a word speeds up processing (e.g., [28]), and most of the semantic information carried by an inflected word form is related to the stem. Third, independent evidence from evoked brain potentials during lexical access suggests that semantic mismatch with the stem affects syntactic mismatch with the suffix, but not the other way round [29]. Fourth, the study of language universals shows a preference of suffixes over prefixes that can reflect computational priority of the stem over the affix [30].

There is controversy with regard to the question whether semantic access takes place only after word form has been processed (e.g., [5]) or in a cascaded manner where the meaning of the word is activated rather early, before the word form analysis has been completed (e.g., [3], [31]). The results from our picture-word matching task cannot be used to disentangle these two alternatives. However, they indicate that when speeded semantic decisions are being made on the basis of the stem vs. suffix/preposition, the stem has a headstart.

As pointed out by Amenta and Crepaldi [32], most current models of morphological processing do not assume a differential role for stems and affixes in visual recognition. It should be noted, however, that many of recent models (e.g., [1], [2], [5], [7]) have primarily focused on derived words and/or earlier levels of processing, and mostly based their interpretations on the visual lexical decision task (with or without masked priming). The current semantic matching task is different in tapping more central levels of processing. One model that does consider this issue, however, is the Naive Discriminative Reader [33] which postulates mappings from orthography (letters, bigrams) to semantics without intervening morpholexical representations. At the semantic level, both stems and affixes are represented, i.e., the NDR model is fully decomposed. Interestingly, the NDR model assigns larger weights to stem meanings than affix meanings in visual word recognition, an implementation that is in line with the present results.

The present results suggest that the mapping from lemma units to semantic representations is stronger for word stems than for affixes. Further studies should still verify our findings with prefixes. Provided that our interpretation holds in further investigations, future models of morphological processing should take into account the primacy of the stem in lexical-semantic access.

## Supporting Information

File S1 **Supporting Information.**
(DOCX)
