# Supplementary material for: Testing the Stem Dominance Hypothesis: Meaning Analysis of Inflected Words and Prepositional Phrases
Source: PLoS One. 2014 Mar 27;9(3):e93136. doi: 10.1371/journal.pone.0093136 (PMC3968051; doi:10.1371/journal.pone.0093136)
Supplement: File S1 — Supporting Information. (DOCX) [file pone.0093136.s001.docx]

**Supporting Information**

*Linear mixed effects analyses for Experiment 1*

The RTs were log-transformed and analyzed using linear mixed models in the R package *lme4* [S1] and *languageR* [S2] after removing data with incorrect responses. We included both participants and items as random effects [S3], and non-match stimulus type as a fixed-effects factor. We checked for normality and homogeneity by visual inspections of plots of residuals against fitted values, and refitted the model after excluding outliers with a standardized residual at a distance greater than 3 standard deviations from zero. This model outperformed the null model that only included the random effects but not the fixed-effects factor, as confirmed by likelihood ratio tests. For monolinguals, the results showed a significant effect of non-match stimulus type (see Table S1), and multiple comparisons of means using Tukey contrasts in the R package *multcomp* [S4] confirmed that the N+P- condition elicited significantly longer RTs than the other two conditions (contrast to N-P+, estimate -0.127, z = -5.33 , *p* < 0.0001; contrast to N-P-, estimate -0.156, z = -6.57, *p* < 0.0001), but the difference between other two non-match stimulus type conditions was not significant (estimate -0.029, z = -1.24, *p* = 0.433).

The error rate analyses were performed by fitting a logistic mixed effects regression model (*lmer* in R with binomial family) with subjects and items as random effects (e.g. [S3, S5]) and non-match stimulus type as a fixed effect. The model revealed a significant effect of non-match stimulus type (see Table S2). Multiple comparisons with Tukey contrasts showed that the N+P- condition elicited significantly more errors than the N-P+ condition or the N-P- condition (for both: estimate 1.707, z = 3.36 , *p* = 0.002), and the latter two did not differ from each other in terms of errors.

*Linear mixed effects analyses for Experiment 2*

For the bilinguals’ data, the linear mixed effects analyses were performed similarly to those of Experiment 1. The results of RTs for *Swedish* showed a significant effect of the non-match stimulus type (see Table S3), and multiple comparisons using Tukey contrasts confirmed that the N+P- condition showed longer RTs than the other conditions (comparison to N-P+, estimate -0.127, z = -5.71, *p* < 0.0001; comparison to N-P-, estimate -0.169, z = -7.58, *p* < 0.0001), but the two other non-match stimulus type conditions did not differ from one another (estimate -0.041, z = -1.87, *p* = 0.148). The error analyses for Swedish showed an effect of non-match stimulus type (Table S4). The N+P- condition elicited more errors than the N-P- condition (estimate 1.271, z = 2.38, *p* = 0.045), but the difference to the N-P+ condition was not significant (estimate 0.789, z = 1.75, *p* = 0.185). The N-P- vs. N-P+ comparison did not show a significant difference either (estimate 0.482, z = 0.81, *p* = 0.696).

The results for RTs in the *Finnish* language task of the bilinguals showed a significant effect of non-match stimulus type (Table S5), with the N+S- condition eliciting longer RTs than the other two conditions (contrast to N-S+, estimate -0.189, z = -8.01, *p* < 0.00001; contrast to N-S-, estimate -0.195, z = -8.27, *p* < 0.00001), which in turn did not differ from one another (estimate -0.006, z = -0.266, *p* = 0.962). The error analyses for Finnish revealed differences between conditions (Table S6): the N+S- condition showed higher error rates than the N-S+ condition (estimate 1.575, z = 3.16, *p* = 0.004) and the N-S- condition (estimate 2.499, z = 3.37, *p* = 0.002). The latter two conditions did not differ in terms of their error rates (estimate 0.924, z = 1.10, *p* = 0.503)

**Table S1**. Results for non-match stimulus type in analysis of reaction times of the monolinguals.

Estimate Std. Error *t-*Value *p*MCMC

(Intercept) 6.73349 0.04205 160.12 0.0001

N-P+ -0.13178 0.02423 -5.44 0.0001

N-P- -0.15356 0.02420 -6.35 0.0001

Reference level: N+P-

**Table S2.** Results for non-match stimulus type in analysis of errors of the monolinguals.

Estimate Std. Error z*-*Value Pr(>|z|)

(Intercept) 2.9250 0.2332 12.542 < 0.0001

N-P+ 1.7073 0.5086 3.357 < 0.001

N-P- 1.7072 0.5086 3.356 < 0.001

Reference level: N+P-

**Table S3**. Results for non-match stimulus type in analysis of reaction times of the bilinguals in the Swedish language task.

Estimate Std. Error *t-*Value *p*MCMC

(Intercept) 6.63547 0.04496 147.6 0.0001

N-P+ -0.12723 0.02229 -5.71 0.0001

N-P- -0.16867 0.02226 -7.58 0.0001

Reference level: N+P-

**Table S4.** Results for non-match stimulus type in analysis of errors of the bilinguals in the Swedish language task.

Estimate Std. Error z*-*Value Pr(>|z|)

(Intercept) 3.2636 0.3039 10.738 < 0.0001

N-P+ 0.7891 0.4517 1.747 0.0806

N-P- 1.2708 0.5340 2.380 0.0173

Reference level: N+P-

**Table S5.** Results for non-match stimulus type in analysis of reaction times of the bilinguals in the Finnish language task.

Estimate Std. Error *t-*Value *p*MCMC

(Intercept) 6.68372 0.04529 147.6 0.0001

N-S+ -0.18872 0.02356 -8.01 0.0001

N-S- -0.19496 0.02357 -8.27 0.0001

Reference level: N+S-

**Table S6.** Results for non-match stimulus type in analysis of errors of the bilinguals in the Finnish language task.

Estimate Std. Error z*-*Value Pr(>|z|)

(Intercept) 2.7422 0.2151 12.747 < 0.0001

N-S+ 1.5752 0.4989 3.157 0.0016

N-S- 2.4994 0.7408 3.374 0.0007

Reference level: N+S-

**References:**

S1. Bates DM, Maechler M, Bolker B, Walker S (2014) lme4: Linear mixed-effects models using

Eigen and S4. R package version 1.0-6.

S2. Baayen RH (2013) languageR: Data sets and functions with "Analyzing Linguistic Data: A

practical introduction to statistics". R package version 1.4.1.

S3. Baayen RH (2008) Analyzing Linguistic Data: A Practical Introduction to Statistics Using R.

Cambridge: Cambridge University Press.

S4. Hothorn T, Bretz F, Westfall P, Heiberger RM, Schuetzenmeister A (2013) multcomp:

Simultaneous inference in general parametric models. R package version 1.3-1.

S5. Jaeger TF (2008) Categorical data analysis: away from ANOVAs (transformations or not) and

towards logit mixed models. J Mem Lang 59: 434–446.

.
